# CMScaller: an R package for consensus molecular subtyping of colorectal cancer pre-clinical models

**DOI:** 10.1038/s41598-017-16747-x

**Published:** 2017-11-30

**Authors:** Peter W. Eide, Jarle Bruun, Ragnhild A. Lothe, Anita Sveen

**Affiliations:** 1 Department of Molecular Oncology, Institute for Cancer Research, Oslo University Hospital, Oslo, NO-0424 Norway; 2 K.G. Jebsen Colorectal Cancer Research Centre, Oslo University Hospital, Oslo, NO-0424 Norway; 3 Institute for Clinical Medicine, University of Oslo, Oslo, NO-0318 Norway

## Abstract

Colorectal cancers (CRCs) can be divided into four gene expression-based biologically distinct consensus molecular subtypes (CMS). This classification provides a potential framework for stratified treatment, but to identify novel CMS-drug associations, translation of the subtypes to pre-clinical models is essential. The currently available classifier is dependent on gene expression signals from the immune and stromal compartments of tumors and fails to identify the poor-prognostic CMS4-mesenchymal group in immortalized cell lines, patient-derived organoids and xenografts. To address this, we present a novel CMS classifier based on a filtered set of cancer cell-intrinsic, subtype-enriched gene expression markers. This new classifier, referred to as CMScaller, recapitulated the subtypes in both *in vitro* and *in vivo* models (551 in total). Importantly, by analyzing public drug response data from patient-derived xenografts and cell lines, we show that the subtypes are predictive of response to standard CRC drugs. CMScaller is available as an R package.

## Introduction

Colorectal cancer (CRC) is the fourth most common cause of cancer deaths worldwide^1^. Gene expression profiling shows promise to identify clinically important subtypes^2–6^, including a mesenchymal-like subgroup with high stromal infiltration, poor patient prognosis^7^ and poor response to standard treatments such as oxaliplatin^8^ and antibodies against EGFR^3,5,9^. Based on gene expression profiles from close to 4000 primary tumors, an expert consortium recently proposed a classification scheme reconciling this work and dividing CRCs into four biologically distinct subtypes^10^. In this consensus system, CMS1-immune comprises most tumors with microsatellite instability (MSI) and is characterized by infiltration of activated immune cells. CMS2-canonical and CMS3-metabolic both show epithelial characteristics, with oncogene amplification and high WNT and MYC signaling predominantly in CMS2 and metabolic reprogramming in CMS3. CMS4 comprises the more mesenchymal-like cancers, with high stromal infiltration and poor patient prognosis^10,11^. So far, identification of subtype-specific drug responses has only scratched the surface, and CMS classification presents an unexploited basis for stratified treatment and drug repurposing in CRC.

*In vitro* and *in vivo* models of CRC recapitulate the molecular profiles of primary cancers^12–15^, as well as clinically relevant pharmacogenomic associations and are suitable tools for drug discovery^16,17^. Publicly available data from pre-clinical drug screen studies represent an invaluable resource, however, analysis of CMS-drug associations is hindered by imprecise subtyping of the models. The original classifier was developed specifically for primary CRCs (pCRC), and it was recently shown that it fails to identify the CMS1-immune and CMS4-mesenchymal groups in cell lines, patient-derived organoids and xenografts (PDX)^15,18–20^, due to the absence of human immune-related signatures, stromal components and extra-cellular matrix in cell cultures and animal models^7,10,18–22^. However, at least some CRC cell lines are mesenchymal-like^5,16,23,24^ and both organoids and PDXs can be established with minimal bias in terms of clinical and molecular covariates of the originating tumors^14,15,25^. This indicates that the apparent lack of CMS4-like models is not a result of biological adaptation to or selection for culturing conditions. We aimed to develop a classifier optimized for pre-clinical models and introduce here CMScaller, an algorithm for CMS classification in the absence of human tumor stroma. We apply CMScaller to *in vitro* and *in vivo* models with publicly available gene expression data and show that it enables pre-clinical analysis of CMS-drug associations.

## Results

### Pre-clinical models require a cancer cell-intrinsic CMS classifier

**Figure 1:**
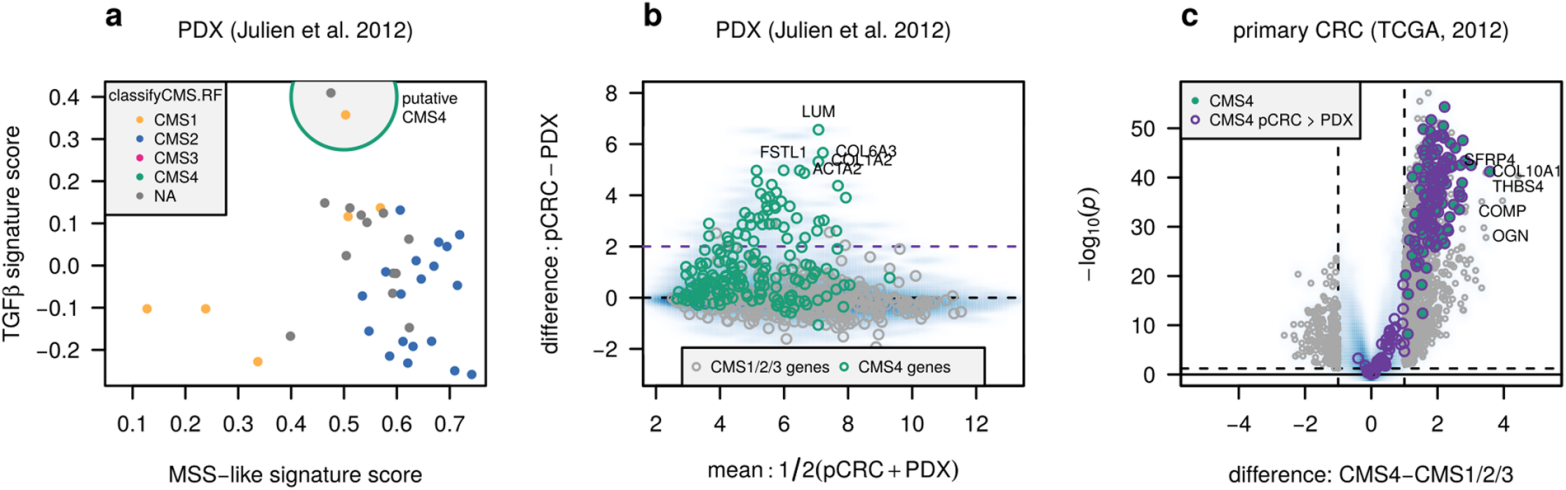
CMS4-mesenchymal markers in primary cancer are partially lost upon xenografting. (**a**) Single-sample gene expression enrichment scores for gene sets of TGFβ responses versus MSS-like characteristics identify two PDX models with particularly strong CMS4 characteristics (encircled). Samples are colored according to CMS predictions based on the original CMSclassifier. (**b**) Differential gene expression between pCRCs and PDX models, plotted against mean overall expression, indicates that genes included as markers in the original CMSclassifier and highly expressed in CMS4 primary tumors (green) show reduced expression in PDXs. The top-5 differentially expressed genes are labeled. Units are log_2_(signal). (**c**) Volcano plot of differential expression analysis of CMS4 versus CMS1/2/3 primary CRCs. Highlighted in purple are the genes differentially expressed between pCRCs and PDXs (absolute LFC > 2). The five genes with the largest absolute difference between CMS4 and CMS1/2/3 are labeled. CMS: consensus molecular subtype; LFC: log_2_fold-change; MSI/MSS: micro-satellite instable/stable; PDX: patient-derived xenograft; pCRC: primary colorectal cancer.

To illustrate why the original CMS classifier is not applicable to pre-clinical models, the classifyCMS.RF function in the R package CMSclassifier^10,26^ was applied to gene expression data from CRC cell lines (*n* = 131)^27^, organoids (*n* = 22 + 26)^15,16^ and PDXs (*n* = 40 + 37 + 51 + 244)^14,20,28,29^. Of all 551 samples, 219 (66%) were unclassified using default parameters. Among the classified, 245 (74%) were CMS2, and only 3 samples (0.9%) were identified as CMS4-mesenchymal (Supplementary Table 1). In one PDX dataset predicted to lack the CMS4 subtype, single-sample gene set expression enrichment analysis indicated strong TGFβ response and MSS-like characteristics in two samples, reminiscent of CMS4 characteristics (Fig. 1a).

To identify genes expressed in or induced by the human tumor microenvironment and therefore with reduced levels in xenotransplants, we performed differential gene expression analysis comparing 30 pCRCs against 40 PDX models^14^. A significant proportion of genes (44/178, 25%) included as features in the original CMS classifier and highly expressed in CMS4 pCRC were reduced or almost completely lost in the PDX samples (*log*_2_fold-change (LFC) > 2, Fig. 1b). Considering all 108 candidate CMS4 genes with reduced expression in the PDXs, the set was significantly enriched for genes involved in biological pathways characteristic of the tumor stroma, including epithelial to mesenchymal transition (EMT), extracellular matrix organization and angiogenesis, which ranked 1^st^, 2^nd^ and 8^th^ in terms of significance among 2038 unselected gene sets from reactome.org^30^ and MSigDB Hallmarks^31^ (hypergeometric test, Supplementary Table 2). Of the 11921 genes overlapping between the pCRC and PDX datasets, 11753 genes showed low differential expression between pCRCs and PDXs (pCRC-PDX LFC < 2, no *p*-value threshold), 921 (7.8%) of these were identified as differentially expressed between CMS4 and CMS1/2/3 in pCRCs (LFC > 1, false discovery rate adjusted-*p* < 0.1), indicating that CMS4 cancer cells present intrinsic gene expression signals that can be used for subtyping (Fig. 1c).

### Development, application and evaluation of the cancer cell-adapted CMScaller

**Figure 2:**
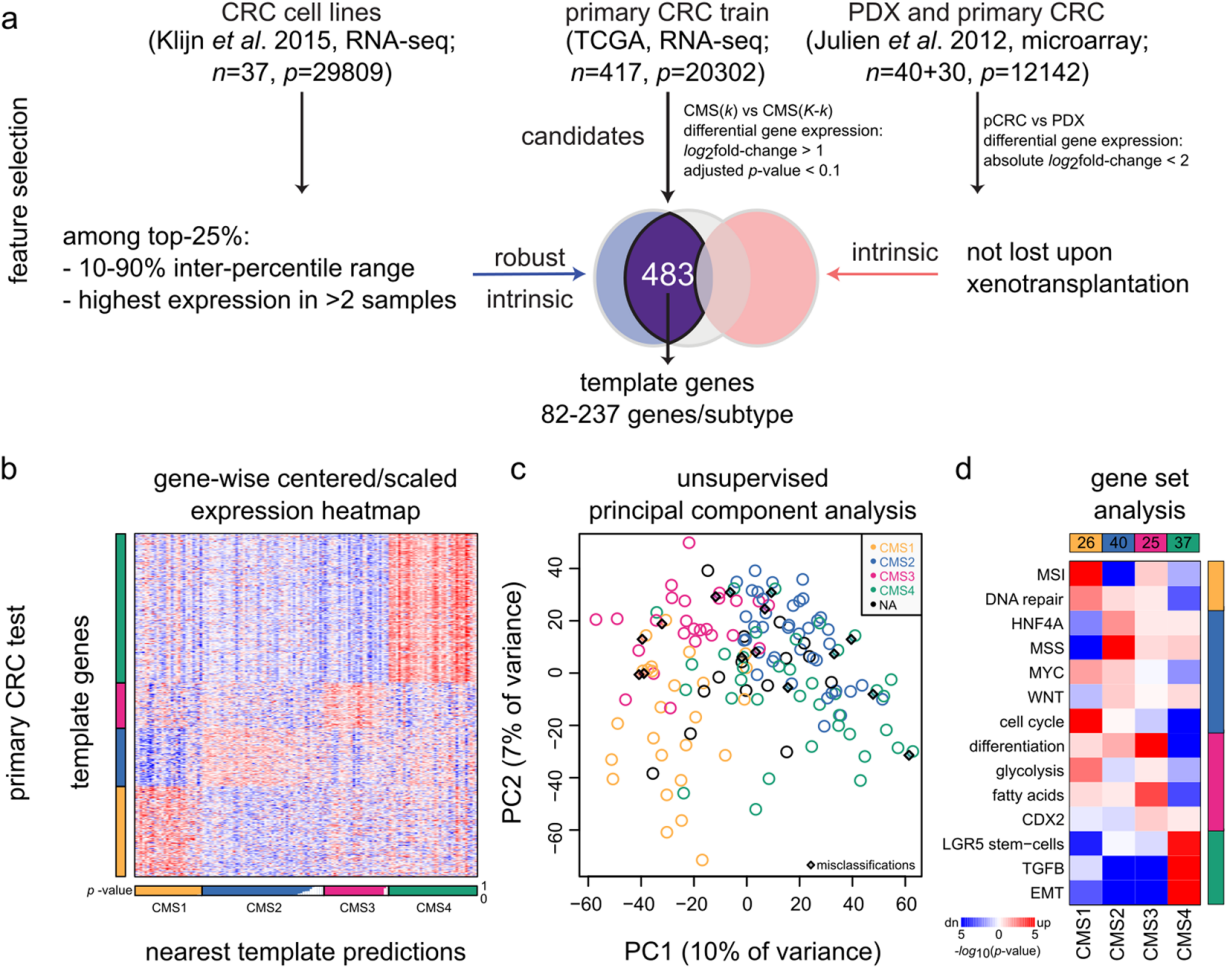
CMScaller feature selection and performance in pCRC. (**a**) Schematic illustration of gene filtering-approach. Three different datasets (top) were used to identify robust cancer cell-enriched subtype markers (only genes represented in all three datasets were considered). (**b**) CMScaller performance on the test set of pCRCs from TCGA (n = 143). Heatmap shows the relative expression levels of subtype marker genes (vertical bar) with classifications indicated below (horizontal bar, white indicate prediction confidence p-values). (**c**) Plot shows results from principal component analysis (expression data batch-adjusted for sequencing-platform). Disagreements between CMScaller and CMSclassifier are indicated with diamonds. (**d**) Heatmap shows results from mRNA gene set analysis, confirming enrichment of known characteristics in each CMS group (details of the gene sets are given in Supplementary Table 3). Red and blue indicate relative up- and down-regulation, respectively, and color saturation represents increasing statistical significance, as indicated. dn: down-regulation; n/p: number of samples/features; NA: not assigned; PC: principal component; PDX: patient-derived xenograft; pCRC: primary colorectal cancer; RNA-seq: RNA-sequencing; TCGA: The Cancer Genome Atlas; up: up-regulation.

Accordingly, to construct a classifier useful for pre-clinical models, we set out to identify cancer cell-intrinsic subtype markers by selecting genes that (i) were highly expressed in each CMS group compared with the rest in pCRCs (LFC > 1, adjusted-*p* < 0.1; representing candidate markers), (ii) had a large range in expression values across a panel of CRC cell lines^32^ (top-25% 10–90 cross-sample inter-percentile range) and were highly expressed in at least a subset (top-25% within-sample read count in at least three cell lines; representing markers that are informative in cancer cells) and (iii) were not lost upon xenotransplantation^14^ (pCRC-PDX LFC < 2, no *p*-value threshold; representing cancer cell-intrinsic markers, Fig. 2a).

The filtered gene set was used as input for nearest template prediction^33^, resulting in the CMScaller algorithm (Fig. 2b). Although created for pre-clinical models, CMScaller performed well also in pCRCs. Using the original CMS classifications as reference for pCRCs from TCGA, the overall prediction accuracy was 0.83 (95% confidence interval 0.74–0.9, *p* = 1.5 × 10^−13^, binomial test for accuracy better than no information rate). Lowest class-wise sensitivity and specificity were found for CMS3 (0.71) and CMS4 (0.91), respectively (Supplementary Table 4). However, gene expression-based principal component analysis showed that most disagreements were at class-boundaries, where the CMS groups are least distinct (Fig. 2c).

Cell lines represent 100% cancer, and the rationale for adding the CRC cell line-filter was to exclude markers of non-carcinoma cell types (genes preferentially expressed by the tumor stroma will not have high expression or expression variation in CRC cell lines). Furthermore, genes with large variation in expression between cell lines are likely to be informative on intrinsic phenotype. To reduce the problem of calling expressed *versus* non-expressed genes, a dataset based on RNA-sequencing (rather than microarray data) was applied. However, to test how sensitive CMScaller is to thresholds and data sets used during development, we generated four additional prediction template gene sets with the following changes: (i) adjusted-*p* threshold for differential expression analysis reduced from 0.1 to 10^–4^ in comparisons among subtypes in pCRC; (ii) selected CRC cell line panel^32^ replaced with another independent RNA sequencing-based dataset^34^; (iii) pCRC-PDX LFC threshold reduced from two to one and (iv) any gene independently reported as lost in PDXs by Isella *et al*. was excluded^7^ (Supplementary Fig. 1a,b). For the four resulting classifiers, the number of genes overlapping with the original CMScaller template set ranged from 205 to 459 (21–95% overlap in features). These 1 + 4 templates were used to perform five independent classifications on the Gao *et al*. PDX models^29^ (Supplementary Fig. 1b,c). The classification concordance ranged from 0.95 to 0.98 and, critically, 47/50 samples (94%) were consistently assigned to a single CMS using all templates (the last sample was consistently unclassified).

We have previously provided CMS classification of widely used, immortalized CRC cell lines, and shown that these *in vitro* models recapitulate the properties of the CMS groups [Sveen submitted]. Here, we demonstrate that CMScaller can robustly classify also CRC organoids and PDXs, and outperforms the original CMSclassifier in seven publicly available datasets, including 26^15^ and 22 organoids^16^, 40^14^, 37^28^, 51^29^ and 244^20^ PDX models, as well as a dataset of 131^27^ cell lines. Using the original CMSclassifier, most samples were classified as CMS2, and only 3/551 as CMS4 (Supplementary Table 1). In contrast, using CMScaller, all CMS groups were found to be present in all datasets and CMS4 accounted for 7.5–22% of the samples (Supplementary Table 5). Notably, 46% of the metastatic CRC-derived PDXs were not assigned any CMS, perhaps due to biased representation of individual CMS groups compared to primary CRCs (ref. Discussion).

**Figure 3:**
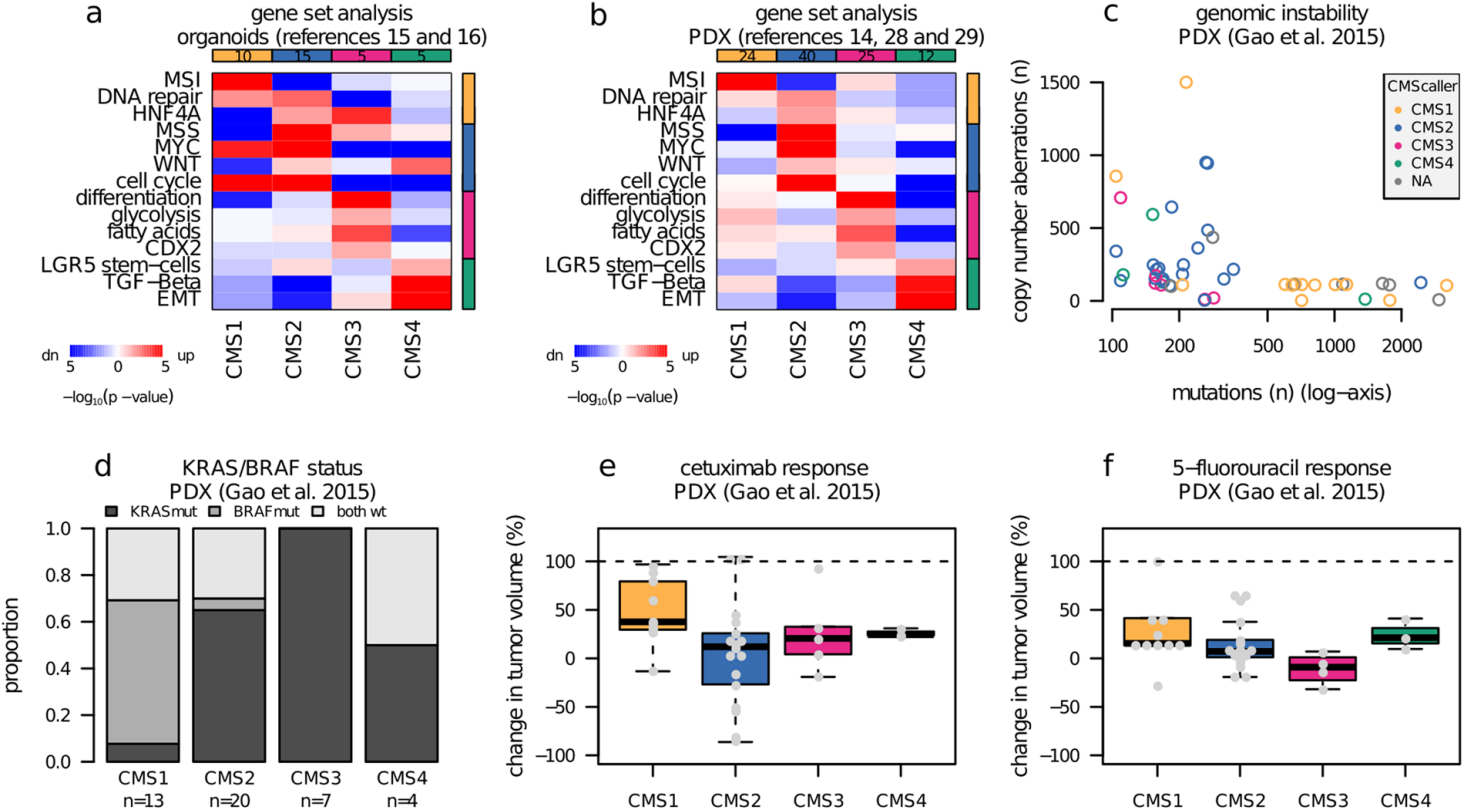
CMS classified PDXs recapitulate relative drug responses observed in patients. (**a**) Heatmap visualization of mRNA gene set analysis showing selected CMS-informative signatures for comparisons of organoids (n = 48) classified with CMScaller. Red and blue indicate relative up- and down-regulation, respectively, and color saturation reflects statistical significance. (**b**) Same as a, but for a merged dataset of PDX models (n = 128). (**c**) Plot showing the number of mutations against the number of copy number aberrations (number of genes affected) per sample. Samples are colored according to CMScaller predictions. Horizontal axis is log-transformed for clarity. (**d**) Barplot showing distribution of *KRAS* and *BRAF* mutations per subtype. (**e**) Response to cetuximab as measured by change in tumor volume (best average response) among PDXs stratified by CMS subtype. (**f**) Response to 5-fluorouracil as measured by change in tumor volume (best average response) among PDXs stratified by CMS subtype. CMS: consensus molecular subtype; mut: mutation; PDX: patient-derived xenograft; wt: wild type

Gene set expression analyses showed that known CMS group associations were recapitulated in both organoids and PDX models (Fig. 3a,b). Specifically, CMS1 was MSI-like, CMS2 was MSS-like, had activated HNF4A, MYC and WNT target gene sets, CMS3 had up-regulated CDX2 targets and metabolic processes, and CMS4 models showed strong relative activation of TGFβ and EMT. The same pattern was not seen for predictions based on the original CMSclassifier (Supplementary Fig. 2). To further support the validity of our subtyping, we analyzed additional molecular data available in two of the datasets, including MSI-status in organoids from Fujii *et al*.^15^ and mutation data in PDXs from Gao *et al*.^29^. In the organoids, in line with expectations, 5/6 MSI samples were predicted to be CMS1-immune (CMS1/MSI: *p* = 1.6 × 10^−4^, Fisher’s exact test). In the PDXs, both the number of total mutations and *BRAF* mutations were significantly higher in CMS1 models (Fig. 3c,d, number of mutations: *p* = 9.9 × 10^−4^, Mann-Whitney test and *BRAF*: *p* = 4× 10^−4^, Fisher’s exact test), and the median number of copy number aberrations was 216 for CMS2, significantly higher than 112 for non-CMS2 (*p* = 5.9× 10^−3^, Mann-Whitney test).

To further evaluate the performance of CMScaller, its prediction accuracy and robustness was compared with the original CMS classifications of pCRCs from TCGA in two ways. First, accuracy was evaluated in relation to sample size by dividing the pCRCs into random subsets (*n* = 1000) of varying sizes (range 10–80 samples). Accuracy distributions indicated that prediction uncertainties were relatively low when sample sizes exceeded more than approximately 40 samples (Supplementary Fig. 3). Second, robustness was evaluated by analyzing whether the CMScaller classifications were “cycle consistent” across the sample types. In other words, we tested whether the Gao *et al*. PDX models (*n* = 51) and assigned CMS groups could be used to derive an independent classifier applicable to the pCRCs (CMS_PDX_ ↔ CMS_CRC_). Using the same strategy as for development of CMScaller, we performed differential expression analysis among the CMS groups to identify subtype markers, this time in PDX models, and filtered out the genes overlapping with the CMScaller template. Overall subtyping accuracy was 0.83 (95% confidence interval 0.74–0.9, *p* = 6.3× 10^−12^, binomial test for accuracy better than no information rate, Supplementary Table 6), indicating that the PDX models and assigned CMS groups represent real biological characteristics of CMS (Supplementary Fig. 4).

### Pre-clinical models recapitulate CMS-associations to standard CRC drugs

Pre-clinical analysis of CMS-drug associations is an important application of CMScaller. To illustrate this potential, we analyzed publicly available drug response data for the standard CRC drugs 5-fluorouracil (5-FU) and cetuximab (anti-EGFR) in 51 CRC PDX models^29^. The Sadanandam *et al*. transit-amplifying subtype, largely corresponding to CMS2, has previously been shown to be particularly sensitive to EGFR-inhibitors *in vitro* ^5,27^, and *in vivo* response has been shown in PDX models with high WNT signaling^18^. We confirm strong *in vivo* response to cetuximab particularly in PDX models predicted to belong to the CMS2 group (*p* = 0.024, CMS2 versus non-CMS2, Mann-Whitney test, Fig. 3e), independent of *KRAS/NRAS/BRAF* mutations (*p* = 0.016, triple wild types only, CMS2 versus non-CMS2, Mann-Whitney test). Importantly, the same association was observed for the dataset of PDXs derived from metastatic CRCs^20^, representing a clinical setting where anti-EGFR treatment is routinely used (*p* = 0.01, OR = 2.9, CMS2 versus non-CMS2 independent of *KRAS/BRAF/NRAS* status, Fisher’s Exact test, Supplementary Fig. 5a). Analysis of CRC cell lines with public gene expression data^27^ and cetuximab response data^35^ (only non-duplicated cell lines with assigned CMS, *n* = 40, Supplementary Fig. 5b) confirmed our previous finding [Sveen submitted] that CMS2 was significantly associated with response to cetuximab also *in vitro* (*p* = 3.4× 10^−3^, OR = 8.3, CMS2 versus non-CMS2, Fisher’s Exact test).

Patients with MSI tumors have been shown to respond poorly to 5-FU^36^, and consistently, 5-FU had poor anti-tumor activity in CMS1-immune PDXs (Fig. 3f). Furthermore, CMS4 also showed a poor response to 5-FU compared with CMS2/3 PDXs (*p* = $0.12$, CMS4 vs CMS2/3 Mann-Whitney test), although the sample number was small (*n*_CMS4_ = 4). For validation, and to eliminate the potential effect of MSI, we analyzed *in vitro* 5-FU response data for a set of MSS CRC cell lines^37^ (only non-duplicated MSS cell lines with assigned CMS, *n* = 34, Supplementary Fig. 6). In CMS1-CMS4 cell lines, the median concentrations required for 50% growth inhibition were 1.1, 1.1, 4.1 and 6.8 µM 5-FU respectively, confirming a poor response in CMS4 (*p* = 7.2 × 10^−3^, CMS4 vs CMS1/2/3 Mann-Whitney test).

## Discussion

Pre-clinical models are invaluable tools for drug discovery, but identification of pharmacogenomic associations depends on accurate molecular subtyping. We report the development and performance of CMScaller, a CMS classifier optimized for pre-clinical model systems of primary colorectal cancer, including cell lines, organoids and xenografts. We illustrate the potential of CMScaller to identify CMS-associated responses to standard CRC drugs, including 5-FU-based chemotherapy, the most widely used oncological treatment regimen in CRC. In patients, treatment is contraindicated in the MSI subtype, but response rates are incomplete also within the MSS group. The PDX and cell line drug response data presented here suggest that the CMS-groups add additional predictive information, indicating a poor response in CMS4. In concordance, previous studies have shown limited benefit from chemotherapy in patients with mesenchymal tumors^38^, also when adding oxaliplatin^8^, and there is mounting evidence that stromal cells confer resistance to chemotherapy^39^. However, these pre-clinical data indicate that the CMS4 group has cancer cell-intrinsic characteristics conferring poor response also in the absence of the tumor microenvironment.

It has been suggested that CMS4 and the related stem-cell/serrated/mesenchymal subtypes mainly reflect tumors dominated by infiltrating stromal cells. Accordingly, novel CRC classifications based on cancer cell-intrinsic gene expression signals were recently proposed^20,40^. There is an interplay between cancer and stromal cells and we hypothesized that the stromal composition in part is determined by cancer-cell intrinsic features. Thus, analogously to how it is reasonable to assume that CMS1 cancer cell-intrinsic immunogenicity may explain the infiltration of activated immune cells in the tumor microenvironment, particular aspects of CMS4 cancer cells may lead to aggressive growth intimately associated with the formation of tumors with abundant cancer-associated fibroblasts and a poor clinical outcome. In a related work, we showed that undifferentiated CRC cell lines, predominately CMS1 and CMS4, had elevated mRNA expression of *TGFB1* and *TGFB2*, encoding TGFβ cytokines^13^. It is therefore tempting to speculate that CMS4 cancer cells through such paracrine signaling are actively remodeling their microenvironments.

CMScaller was developed to provide robust classification across gene expression platforms. This is demonstrated by gene set analyses recapitulating the hallmarks of each CMS group in datasets generated on different technological platforms and for different biological sample types. Robustness can in part be ascribed to the fact that gene expression is highly co-regulated and, despite of thousands of features, informationally surprisingly low-dimensional^41^. Consequently, missing, noisy or erroneous measurements are compensated for by other co-regulated genes in the nearest template prediction algorithm applied^33^. This may also explain why the tested changes to the template gene set had little impact on the resulting sample classifications.

Although the template genes were selected to enrich for cancer-cell intrinsic signals, they are not exclusively expressed by cancer cells. For example, the CMS4 marker *VIM* is expressed in fibroblasts, but have higher expression among CMS4 cancer cells than CM2–3 and is therefore a useful template gene. Importantly, stricter gene filtering for stromal expression had limited impact on the resulting classifications. Still, CMScaller in its current implementation is not recommended for use with samples with a different human stromal component than primary CRCs, including *e*.*g*. patient biopsies of metastatic CRC. Another inherent limitation to CMScaller is that the input gene expression data should be centered and scaled. The implication is that small datasets inescapably introduce prediction uncertainty, due to the potential for biased representation of either subtype. Our estimations indicate that this is a concern for datasets with fewer than approximately forty samples, but becomes minor when *n* exceeds this limit. Similarly, caution is warranted in highly selected datasets where molecular distributions are expected, or known, to severely deviate from pCRC cohorts, including metastatic samples^42^. Importantly, for both organoids and PDXs, it has been shown that the models recapitulate the heterogeneity of their original tumors^14,15,25^. However, to assess potential bias, we recommend analyzing CMS-associations of additional molecular markers, for instance MSI-status and *BRAF* mutations. Additionally, CMScaller includes a function for downstream gene set expression analyses, allowing for assessment of CMS hallmarks.

Within its proper context of pCRC-derived cohorts, accuracy assessment of CMScaller indicated that prediction errors were mainly made at the class boundaries, where expression patterns are less distinct. This may be explained by intra-tumor heterogeneity^43,44^, both from immune/stromal infiltration (tumor cell percentage and stromal composition) and intrinsic to the cancer cells^45^. The extent of heterogeneity of molecular subtyping has recently been illustrated by single cell RNA-sequencing^46^. We have tested CMScaller on these data, and fair correspondence in CMS group assignments was obtained for individual cancer cells from the same patient. However, with only ten patients, the number was too low to draw any strong conclusions whether CMScaller is useful with such data inputs. From a technical perspective, single-cell RNA-sequencing data is exceedingly noisy and a classifier should be optimized for and take advantage of the digital nature of such data. We envision implementing this as a future option in the CMScaller package, as well as to further improve the prediction template gene set taking advantage of larger, higher-quality datasets as they become available.

In addition to pre-clinical drug response studies, model systems may also be useful for functional analyses of central CMS-associated characteristics in controlled environments. The CMScaller presented here should enable the matching of patient subtypes with appropriate models, and we hope this will be a useful tool for the research community. CMScaller is platform independent and available as an R package.

## Methods

### Gene expression and drug response data

Klijn *et al*. colorectal, liver and stomach cancer cell line mRNA and non-coding RNA-sequencing counts were downloaded from ref.^32^, non-Entrez features were discarded and pre-processing was performed by conditional quantile normalization and variance stabilization using cqn^47^ and DESeq2^48^. Gao *et al*. PDX RNA-sequencing FPKM values were retrieved from Supplementary Table 1 in ref.^29^. GSE35144^28^, GSE64392^16^, GSE74843^15^ and E-MTAB-991^14^ PDX/organoid microarray gene expression datasets were downloaded from GEO^49^/ArrayExpress^50^ and CEL files were pre-processed using the justRMA function in the R package affy^51^, with brainarray Entrez v20 CDFs^52^. Preprocessed cell line and PDX Illumina BeadArray data were downloaded from GSE59857^27^ and GSE76402^20^, *log*_2_transformed and quantile normalized. TCGA level 3 RSEM gene-level RNA-sequencing data^53^ was downloaded from Broad GDAC Firehose [doi:10.7908/C11G0KM9]. For all datasets, non-CRC, neuro-endocrine cancers and same-patient duplicates were discarded prior to analysis. Gao *et al*. PDX drug response data is from ref.^29^ Supplementary Table 1. Isella *et al*. PDX cetuximab response data is from GSE76402. Cell line MSI-status and 5-FU response data were retrieved from Supplementary Tables 1,2 in ref.^37^ and cetuximab response groups from Fig. 2 in ref.^35^.

### Gene expression analysis

Single sample gene set enrichment analysis was performed with the R package GSVA^54^. Differential gene expression analysis was performed using limma^55^. For RNA-sequencing data, voom transformation with quantile normalization was performed prior to the limma modeling^56^. Principal component analysis was performed using the 1000 genes with the largest 10–90% inter-percentile range in expression values as input. RNA-sequencing data was first *log*_*2-*_transformed and batch adjusted according to sequencing platform (HiSeq/GAIIx) using the ComBat method implemented in the R package sva^57^.

### Gene set tests

For CMS marker genes down-regulated in the pCRC *versus* PDX comparison, statistical enrichment among 2038 unselected gene sets retrieved from reactome.org^30^ and MSigDB Hallmarks^31,58^ (v5.2) was assessed assuming a hypergeometric distribution using the phyper function in the R package stats^26^. Camera gene set analysis^59^ and visualization was performed using the R package limma with default parameters and implemented in the CMScaller function CMSgsa. For the latter analysis, gene sets were pre-selected to be likely CMS-informative based on Guinney *et al*. and are listed in Supplementary Table 3 ^10^.

### CMScaller template feature selection

TCGA primary CRC (COADREAD) CMS labels were retrieved from Sage Bionetworks Synapse (syn4978511)^10^. TCGA pCRC RNA-sequencing samples^53^ were randomly assigned to a training (75%) and test set (25%) using the R function sample. Differential expression analysis in the TCGA test set was used to identify *candidate markers* with higher expression in each subtype compared to the remaining samples (LFC > 1, adjusted-*p* < 0.1). To generate the final template gene set, these candidates markers were enriched for cancer cell-intrinsic expression signals according to the following criteria. RNA-sequencing data for CRC cell lines^32^ was used to identify *robust and intrinsic markers*, genes among the top-25% with (i) highest expression in at least three samples and (ii) largest 10–90% inter-percentile range in expression values. limma differential expression analysis comparing pCRC with PDX models^14^ was used to further enrich for *intrinsic markers* defined as genes with LFC < 2. The intersection of candidate, robust and intrinsic markers were used as template features for nearest template prediction^33^, which is a correlation-based algorithm developed to provide robust class prediction for high-dimensional, noisy gene expression data, and which has been successfully adopted to various similar classification tasks (*e*.*g*. ref.^27^).

### CMS classification

CMS classifications were performed using either the original classifyCMS.RF function with default settings in the R package CMSclassfier,^10^ or the novel CMScaller. With CMScaller, prediction confidence is estimated from gene resampling (*n* = 1000) and samples with false discovery rate adjusted *p*-value > 0.05 were “not assigned” (NA). To assess prediction variance, CMScaller was applied on 8 × 1000 random TCGA train subsets (*n* = {10, 20, …, 80}). To determine whether subtyping is sample-type “cycle consistent”, differential expression analysis of PDXs classified by CMScaller was used to develop new templates for nearest template prediction of the TCGA pCRC test set. Genes used for the PDX classification were not included in this new template.

### Additional statistical analysis

For differential gene expression analysis, hypergeometric gene set tests and nearest template predictions, Benjamini-Hochberg false discovery rate adjustment implemented in the R package stats in the function p.adjust was used to account for multiple-testing^26,60^. All reported accuracy values are overall accuracies (the number of classification agreements divided by the number of cases with those not assigned (NA) excluded).

### Data availability

All data analyzed during the current study were retrieved from public sources. In brief, GSE59857, GSE76402, GSE35144, GSE64392 and GSE74843 gene expression datasets were downloaded from Gene Expression Omnibus (GEO). TCGA pCRC gene expression data was downloaded from Broad GDAC Firehose accession doi:10.7908/C11G0KM9. Julien *et al*. PDX and pCRC gene expression dataset E-MTAB-991 was downloaded from ArrayExpress. Additional data was retrieved as described in the relevant Methods sections.

### Code availability

The CMScaller (v0.99.1) R package is available in Supplementary Materials and will be submitted to Bioconductor^61^. Updates will be available on https://github.com/Lothelab/CMScaller. Instructions for installation and example code are given in Supplementary Tables and Figures.

## Electronic supplementary material

Supplementary Tables and Figures

CMScaller
