# Supplementary material for: CMScaller: an R package for consensus molecular subtyping of colorectal cancer pre-clinical models
Source: Sci Rep. 2017 Nov 30;7:16618. doi: 10.1038/s41598-017-16747-x (PMC5709354; doi:10.1038/s41598-017-16747-x)
Supplement: Supplementary file 1 — Supplementary Tables and Figures [file 41598_2017_16747_MOESM1_ESM.pdf]

CMScaller: an R package for consensus molecular subtyping of colorectal cancer pre-clinical models

Supplementary Tables and Figures

Peter W. Eide[1,2,3], Jarle Bruun[1,2], Ragnhild A. Lothe[1,2,3], Anita Sveen[1,2,*]

[1] Department of Molecular Oncology, Institute for Cancer Research and [2] K.G.Jebsen Colorectal Cancer Research Centre, Oslo University Hospital, Oslo, NO-0424, Norway[3] Institute for Clinical Medicine, University of Oslo, Oslo, NO-0318, Norway, *correspondence to anita.sveen@rr-research.no

Table 1: Pre-clinical models classified using classifyCMS.RF[1] with default parameters (center=TRUE, posterior p>0.5). TCGA primary CRC dataset[2] is included for reference and excluded from the subtotals. NA indicates samples not assigned to any CMS. NA: not assigned; PDX: patient-derived xenograft.

Reference	Model	CMS1	CMS2	CMS3	CMS4	NA	Sum
TCGA (2012)	primary CRC (test set)	12	49	15	23	44	143
Medico et al. (2015)	cell lines	16	46	2	1	66	131
van de Wetering et al. (2015)	organoids	3	10	0	0	9	22
Fujii et al. (2016)	organoids	5	15	1	0	5	26
Julien et al. (2012)	PDX	7	19	0	0	14	40
Uronis et al. (2012)	PDX	1	17	7	1	11	37
Gao et al. (2015)	PDX	10	19	5	1	16	51
Isella et al. (2017)	PDX (metatstatic CRC)	26	119	1	0	98	244
	total (excluding primary CRC)	68	245	16	3	219	551

Table 2: Top-10 gene sets in terms of overrepresentation of 108 genes reduced or lost upon upon xenografting (primary CRC-PDX LFC<2). Genes column represents the number of genes in the gene set. Intersect shows the number of genes overlapping between the gene set and the 108 genes reduced/lost in xenografts. FDR represents the false discovery rate adjusted p-values from hypergeometric tests. A total of 2038 gene sets from reactome.org[3] and MSigDB Hallmarks[4] were tested. Only gene set genes among the 11921 genes overlapping between the PDX and pCRC datasets were considered. LFC: log_2fold-change; pCRC: primary colorectal cancer; PDX: patient-derived xenograft.

	genes (n)	intersect (n)	FDR
Hallmark Epithelial Mesenchymal Transition	193	36	<2e-16
Extracellular matrix organization	258	30	<2e-16
Hallmark Coagulation	136	16	5e-11
Regulation of Insulin-like Growth Factor (IGF) transport…	109	13	8e-09
Assembly of collagen fibrils and other multimeric structures	51	10	9e-09
Post-translational protein phosphorylation	93	12	1e-08
Collagen chain trimerization	32	8	9e-08
Hallmark Angiogenesis	36	8	2e-07
Collagen formation	74	10	2e-07
Collagen biosynthesis and modifying enzymes	53	8	4e-06

Table 3: Signatures used for gene set analysis. Columns contains gene set names, references, PubMed identifiers (pmid) and additional information. Genes sets are available as an R object in the CMScaller pakage, geneSets.CMS (named list with vectors of Entrez ids). For TGF-Beta, limma differential expression analysis was used to identify genes >3-fold upregulated after TGFβ treatment. EMT; epithelial-mesenchymal transition; MSI/MSS: micro-satellite instable/stable.

gene set	reference	PubMed ID	source
CDX2	Liu 2007; Liberzon 2011	16990345; 21546393	MSigDB C2 db (v5.2)
Cell cycle (E2F Targets)	Liberzon 2011	21546393	MSigDB Hallmark db (v5.2)
DNA repair	Liberzon 2011	21546393	MSigDB Hallmark db (v5.2)
EMT	Liberzon 2011	21546393	MSigDB Hallmark db (v5.2)
Fatty acids	Croft 2014	24243840	reactome db (accessed 20161212)
Gastro-intestinal markers	Uhlèn 2015	25613900	proteinatlas.org (accessed 20160427)
Glycolysis	Liberzon 2011	21546393	MSigDB Hallmark db (v5.2)
HNF4A	Lucas 2005; Liberzon 2011	16007190; 21546393	MSigDB C2 db (v5.2)
LGR5	Merlos-Suarez 2011	21419747	Supplementary Table 5
MSI	Watanabe 2006; Liberzon 2011	17047040; 21546393	MSigDB C2 db (v5.2)
MSS	Watanabe 2006; Liberzon 2011	17047040; 21546393	MSigDB C2 db (v5.2)
MYC	Liberzon 2011	21546393	MSigDB Hallmark db (v5.2)
TGF-Beta	Fessler 2016	27221051	GSE79461
WNT	Vermeulen 2010	20418870	Supplementary Table 1

Table 4: CMScaller class-wise performance on test set of primary colorectal cancers. Reference labels are from Guinney et al. (2015). CMScaller predictions with false discovery rate adjusted-p>0.05 were set to NA. Table was prepared using the confusionMatrix function in the R package caret[5].

	Class: CMS1	Class: CMS2	Class: CMS3	Class: CMS4
Sensitivity	1.00	0.78	0.71	0.91
Specificity	0.94	0.98	0.96	0.91
Balanced Accuracy	0.97	0.88	0.84	0.91

Table 5: Pre-clinical models classified using CMScaller with default parameters. TCGA primary CRC dataset[2] is included for reference and excluded from the subtotals. CMScaller predictions with false discovery rate adjusted-p>0.05 were set to NA. NA: not assigned; PDX: patient-derived xenograft

Reference	Model	CMS1	CMS2	CMS3	CMS4	NA	Sum
TCGA (2012)	primary CRC (test set)	26	40	25	37	15	143
Medico et al. (2015)	cell lines	16	37	17	29	32	131
van de Wetering et al. (2015)	organoids	5	8	2	3	4	22
Fujii et al. (2016)	organoids	5	7	3	2	9	26
Julien et al. (2012)	PDX	7	15	7	3	8	40
Uronis et al. (2012)	PDX	3	9	9	6	10	37
Gao et al. (2015)	PDX	13	20	7	4	7	51
Isella et al. (2017)	PDX (metatstatic CRC)	28	40	31	32	113	244
	total (excluding primary CRC)	77	136	76	79	183	551

Table 6: Class-wise performance on test set of primary colorectal cancers with templates derived from PDX models. Reference labels are from reference 1. CMScaller predictions with false discovery rate adjusted-p>0.05 were set to not assigned (NA). Table was prepared using the confusionMatrix function in the R package caret[5]. Additional information is included in Supplementary Figure 1.

	Class: CMS1	Class: CMS2	Class: CMS3	Class: CMS4
Sensitivity	1.00	0.91	0.71	0.65
Specificity	0.94	0.88	0.95	0.99
Balanced Accuracy	0.97	0.89	0.83	0.82

a

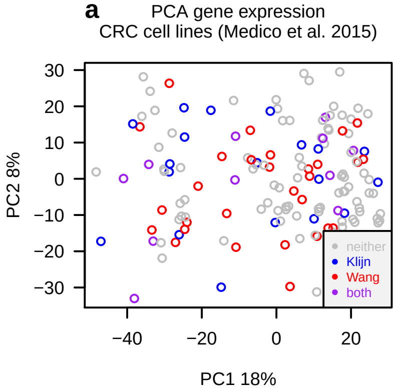

b

Template genes

	added (n)	removed (n)
CellPanel	473	278
Pstrict	0	24
Xstrict	0	78
Istrict	473	278

c

Classification concordance
PDX (Gao et al. 2015)

	concordance
CMScaller-CellPanel	0.95
CMScaller-Pstrict	0.98
CMScaller-Xstrict	0.98
CMScaller-Istrict	0.95

47/50 (94%) concordant/classified (n=51)

Figure 1: **Classifications are robust to changes in template filtering.** **(a)** Principal component analysis of gene expression data shows that both Wang[6] and Klijn[7] datasets present representative samples of CRC cell lines in general. Each point represents a cell line. CRC cell lines included in the Klijn 2015 (CMScaller default), Wang 2017 or both or neither are indicated with colors. Gene expression data is GSE59857 from reference 8. Standardized expression values for top-1000 genes with the largest 10-90% inter-percentile range in signal values were used as input. **(b)** Table shows how many genes are added and removed by changes in template generation; CellPanel: Klijn CRC cell line panel[7] replaced with Wang dataset[6]; Pstrict: pCRC differential expression analysis adjusted-p threshold reduced from 10^{-1} to 10^{-4}; Xstrict: pCRC-PDX differential expression analysis LFC threshold reduced from two to one (iv) Istrict: genes independently reported as lost in PDXs were removed[9] **(c)**. Classification concordance (accuracy) for CMScaller using default and the modified templates for PDX[10] dataset. Concordant refers to the number of samples in agreement for all five templates, classified the number of samples assigned CMS and total is the number of samples considered. LFC: log_2fold-change; pCRC: primary colorectal cancer; PDX: patient-derived xenograft.

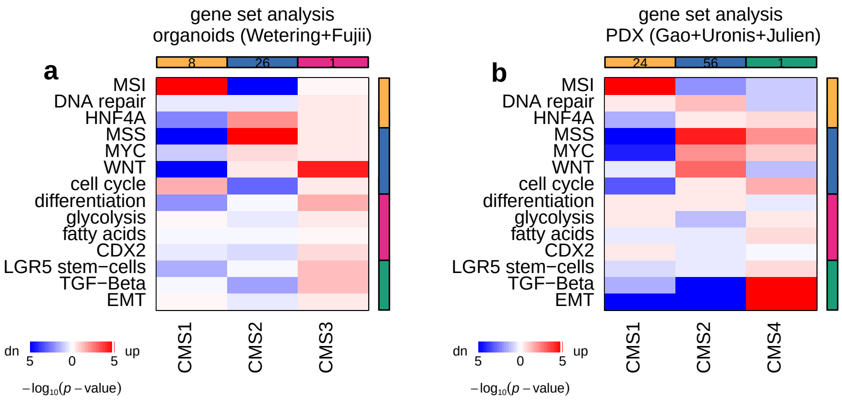

Figure 2: **Gene set analysis shows that differences between CMS organoids** (a) **and PDX** (b) **subtypes are poorly recapitulated using pCRC CMS classifier.** Red and blue indicate relative up- and down-regulation, respectively, and color saturation represents increasing statistical significance, as indicated. Numbers above heatmap indicate predicted samples per subtype.

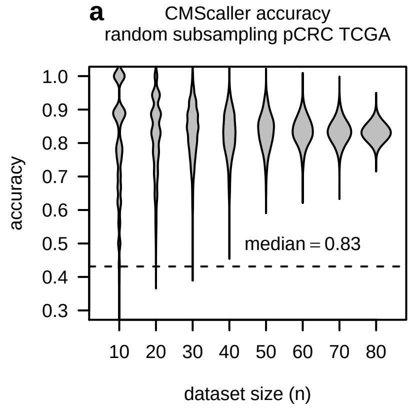

Figure 3: **Prediction accuracy variance is a function of dataset size.** Beanplot shows accuracy distributions for 1000 random TCGA[2] train subsets of size *n*. Horizontal line indicates the no information rate (proportion belonging to largest group, CMS2).

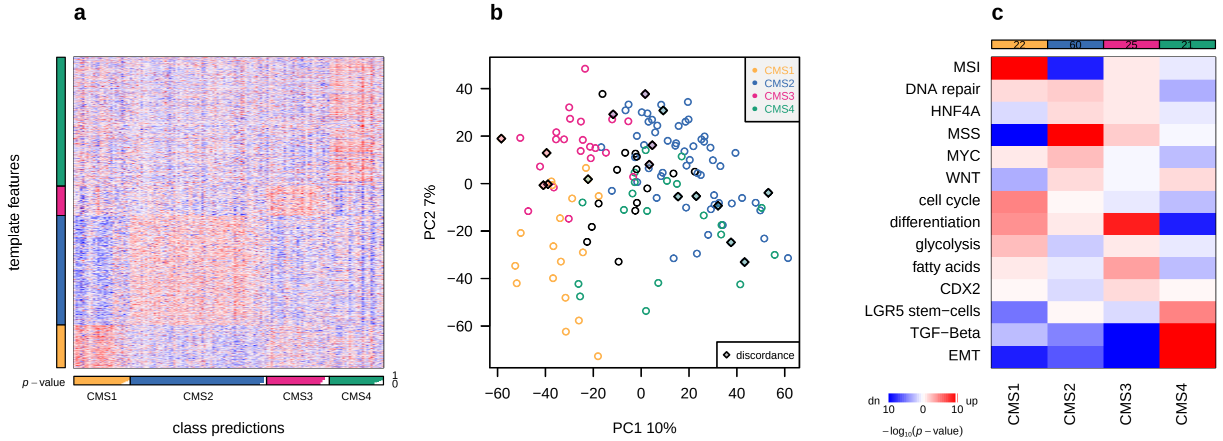

Figure 4: **Primary colorectal cancers CMS classified using templates defined from CMScaller PDXs demonstrate cycle consistency.** In brief, first, Gao *et al.* PDXs[10] were classified using CMScaller. Then, based on these PDXs, differential expression analysis was used to make new prediction templates after excluding genes included in CMScaller. Finally, this PDX-derived template was used to classify test set TCGA primary CRCs[2] and performance assessed by comparing assigned CMS with labels from Guinney *et al.*[1]. Additional information is included in Supplementary Table 6. PDX; patient-derived xenograft; TCGA; The Cancer Genome Atlas.

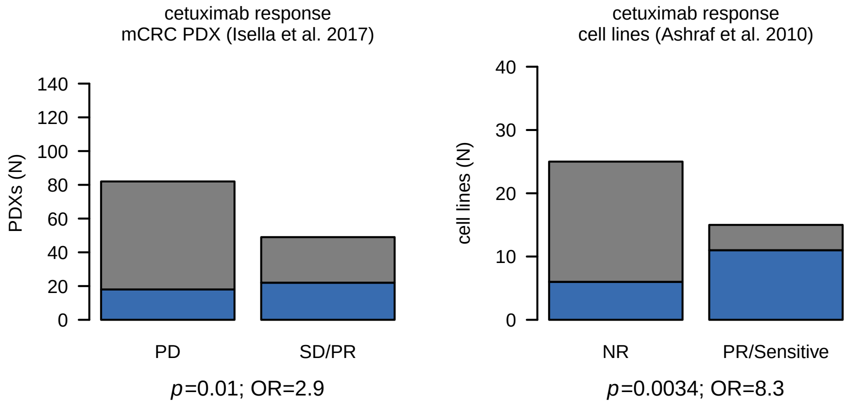

Figure 5: **CMS is a predictor of cetuximab (anti-EGFR) response in colorectal cancer models**. Cetuximab response stratified by CMS2/non-CMS2 (blue/gray, respectively). **(a)** mCRC PDX drug response is from reference 11 where PD is progressive disease and SD/PR stands for stable disease/partial response. **(b)** CRC cell line drug response data is from reference 12. NR is no-response and PR/sensitive indicates cell lines showing partial response or sensitivity towards cetuximab. *p*-values and odds ratios (OR) are from Fisher's exact tests. mCRC: metastatic colorectal cancer; PDX: patient-derived xenograft.

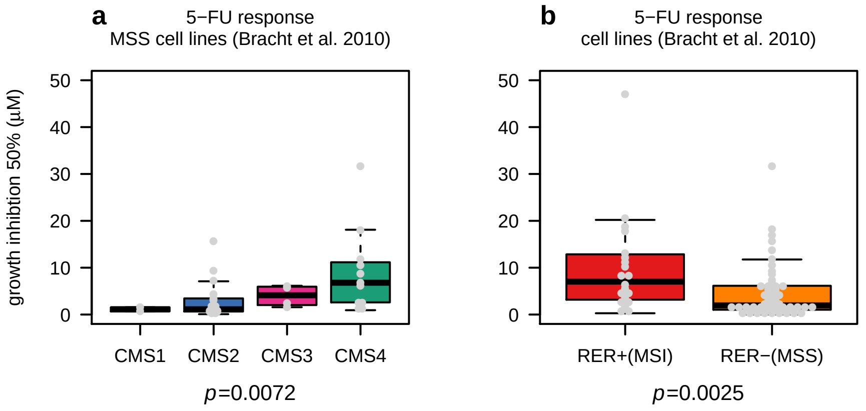

Figure 6: **CMS is a predictor of 5-fluorouracil (5-FU) response in colorectal cancer cell lines**. (**a**) 5-FU response stratified by CMS. (**b**) 5-FU response stratified by MSI-status. Drug response data and MSI status are from Bracht *et al*[13]. Cell line duplicates derived from the same patient were excluded. Higher concentrations indicate higher 5-FU resistance. *p*-values for CMS (CMS4 vs CMS2/3) and MSI are from Mann-Whitney tests. MSI/MSS: micro-satellite instable/stable; RER: replicative error.

Quick start

Code below shows how to install and run CMScaller with included example TCGA colorectal cancer RNA-sequencing data. Function documentation is available through the standard `?function` interface *e.g.* `?CMScaller`. Package updates will be available from https://github.com/Lothelab.

```
################################################################################
# install Bioconductor dependencies - internet connection required
# crcTCGAsubset is an ExpressionSet of TCGA colorectal cancers subset
################################################################################
# dependencies: run if not already installed
# source("https://bioconductor.org/biocLite.R")
# biocLite(c("Biobase", "limma"))
# R package included as Supplementary Material must by unzipped to .tar.gz
# install.packages("pathToPackageFile/CMScaller_0.99.1.tar.gz", repos = NULL)
library(Biobase)
library(CMScaller)
par(mfrow=c(1,3))
# load and show example data (Entrez row names)
emat <- exprs(crcTCGAsubset)
head(emat[,1, drop=FALSE],5)
```

```
##           TCGA-4N-A93T-01A-11R
## 100133144                    9
## 10431                     2450
## 57714                      375
## 645851                      43
## 652919                       0
```

```
res <- CMScaller(emat, RNAseq=TRUE, doPlot=TRUE)
```

```
## performing log2-transform and quantile normalization...
## cosine correlation distance
## 92 samples; 4 classes; 82-237 features/class
## serial processing; 1000 permutation(s)...
## predicted samples/class (FDR<0.05)
##
## CMS1 CMS2 CMS3 CMS4 <NA>
##   14   23   15   28   12
## 12/92 samples set to NA
```

```
cam <- CMSgsa(emat, class=res$prediction, RNAseq=TRUE)
```

```
## 12 samples with class or batch NA's excluded
```

```
# additional examples and technical details
vignette("CMScaller")

################################################################################
# update to latest development version
################################################################################
# install.packages("devtools")
# devtools::install_github("Lothelab/CMScaller")
```

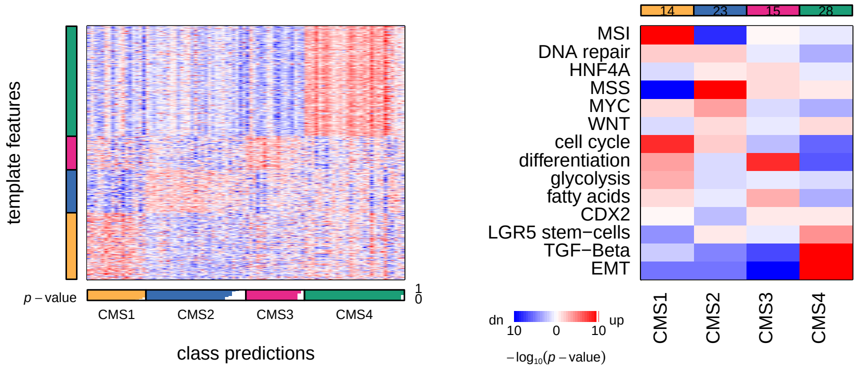

Figure 7: **Graphic outputs for the functions CMScaller() and CMSgsa().**

R packages

Table 7: Version numbers for R packages.

package	version	date	source
annotate	1.54.0	2017-05-12	Bioconductor
AnnotationDbi	1.38.2	2017-10-14	Bioconductor
Biobase	2.36.2	2017-05-12	Bioconductor
BiocGenerics	0.22.1	2017-10-14	Bioconductor
BiocParallel	1.10.1	2017-09-19	Bioconductor
CMScaller	0.99.1	2017-11-21	Scientific Reports
CMSclassifier	1.0.0	2017-05-12	Github (Sage-Bionetworks/CMSclassifier@ba02ec5)
genefilter	1.58.1	2017-05-12	Bioconductor
gplots	3.0.1	2016-03-30	CRAN (R 3.4.1)
graph	1.54.0	2017-05-12	Bioconductor
GSEABase	1.38.2	2017-10-14	Bioconductor
GSVA	1.24.2	2017-10-14	Bioconductor
IRanges	2.10.5	2017-10-14	Bioconductor
limma	3.32.10	2017-10-14	Bioconductor
plotrix	3.6-6	2017-08-16	CRAN (R 3.4.1)
S4Vectors	0.14.7	2017-10-14	Bioconductor
sva	3.24.4	2017-10-14	Bioconductor

Packages explicitly loaded include beanplot[14], Biobase[15], devtools[16], caret[5], CMSclassifier[1], genefilter[17], gplots[18], GSVA[19], IRanges[20], knitr[21], limma[22], plotrix[23], RColorBrewer[24] and sva[25].

References

1. Guinney, J. *et al.* The consensus molecular subtypes of colorectal cancer. *Nat Med* **21,** 1350–1356 (2015).

2. TCGA. Comprehensive molecular characterization of human colon and rectal cancer. *Nature* **487,** 330–337 (2012).

3. Croft, D. *et al.* The Reactome pathway knowledgebase. *Nucl Acids Res* **42,** D472–D477 (2014).

4. Liberzon, A. *et al.* The Molecular Signatures Database Hallmark Gene Set Collection. *Cell Systems* **1,** 417–425 (2015).

5. Wing, M. K. C. from J. *et al. Caret: Classification and Regression Training.* (2015).

6. Wang, J. *et al.* Colorectal Cancer Cell Line Proteomes Are Representative of Primary Tumors and Predict Drug Sensitivity. *Gastroenterology* **153,** 1082–1095 (2017).

7. Klijn, C. *et al.* A comprehensive transcriptional portrait of human cancer cell lines. *Nat Biotech* **33,** 306–312 (2015).

8. Medico, E. *et al.* The molecular landscape of colorectal cancer cell lines unveils clinically actionable kinase targets. *Nat Commun* **6,** (2015).

9. Isella, C. *et al.* Stromal contribution to the colorectal cancer transcriptome. *Nat Genet* **47,** 312–319 (2015).

10. Gao, H. *et al.* High-throughput screening using patient-derived tumor xenografts to predict clinical trial drug response. *Nat Med* **21,** 1318–1325 (2015).

11. Isella, C. *et al.* Selective analysis of cancer-cell intrinsic transcriptional traits defines novel clinically relevant subtypes of colorectal cancer. *Nat Commun* **8,** ncomms15107 (2017).

12. Ashraf, S. Q. *et al.* Direct and immune mediated antibody targeting of ERBB receptors in a colorectal cancer

cell-line panel. *PNAS* **109,** 21046–21051 (2012).

13. Bracht, K., Nicholls, A. M., Liu, Y. & Bodmer, W. F. 5-Fluorouracil response in a large panel of colorectal cancer cell lines is associated with mismatch repair deficiency. *Br J Cancer* **103,** 340–346 (2010).

14. Kampstra, P. Beanplot: A Boxplot Alternative for Visual Comparison of Distributions. *J Stat Softw* **28,** 1–9 (2008).

15. Huber, W. *et al.* Orchestrating high-throughput genomic analysis with Bioconductor. *Nat Meth* **12,** 115–121 (2015).

16. Wickham, H. & Chang, W. *Devtools: Tools to Make Developing R Packages Easier.* (2017).

17. Gentleman, R., Carey, V., Huber, W. & Hahne, F. *Genefilter: Genefilter: Methods for filtering genes from high-throughput experiments.* (2017).

18. Warnes, G. R. *et al. Gplots: Various R Programming Tools for Plotting Data.* (2016).

19. Hänzelmann, S., Castelo, R. & Guinney, J. GSVA: Gene set variation analysis for microarray and RNA-Seq data. *BMC Bioinformatics* **14,** 7 (2013).

20. Lawrence, M. *et al.* Software for Computing and Annotating Genomic Ranges. *PLoS Comp Biol* **9,** e1003118 (2013).

21. Xie, Y. *Dynamic Documents with R and knitr, Second Edition.* (Chapman; Hall/CRC, 2015).

22. Ritchie, M. E. *et al.* Limma powers differential expression analyses for RNA-sequencing and microarray studies. *Nucl Acids Res* **43,** e47 (2015).

23. Lemon, J. Plotrix: A package in the red light district of R. *R-News* **6,** 8–12 (2006).

24. Neuwirth, E. *RColorBrewer: ColorBrewer Palettes.* (2014).

25. Leek, J. T. *et al.* Tackling the widespread and critical impact of batch effects in high-throughput data. *Nat Rev Genet* **11,** 733–739 (2010).
