# Supplementary material for: CMScaller: an R package for consensus molecular subtyping of colorectal cancer pre-clinical models
Source: Sci Rep. 2017 Nov 30;7:16618. doi: 10.1038/s41598-017-16747-x (PMC5709354; doi:10.1038/s41598-017-16747-x)
Supplement: Supplementary file 2 — CMScaller [file 41598_2017_16747_MOESM2_ESM.zip › CMScaller_0.99.1.tar.gz!/CMScaller/inst/doc/CMScaller.pdf]

CMScaller: an R package for consensus molecular subtyping of colorectal cancer pre-clinical models

Peter W. Eide[1,2,3], Jarle Bruun[1,2], Ragnhild A. Lothe[1,2,3], Anita Sveen[1,2]

[1] Department of Molecular Oncology, Institute for Cancer Research and [2] K.G.Jebsen Colorectal Cancer Research Centre, Oslo University Hospital, Oslo, NO-0424, Norway[3] Institute for Clinical Medicine, University of Oslo, Oslo, NO-0318, Norway

- contact: peteid@rr-resarch.no or ansvee@rr-research.no
- Date: 2017-11-21
- package: CMScaller 0.99.1

1 Introduction

Colorectal cancers (CRCs) can be divided into four gene expression-based biologically distinct consensus molecular subtypes (CMS) [1]. This classification provides prognostic stratification of the patients and presents a potential basis for stratified treatment. The original CMS classifier is dependent on gene expression signals from the immune and stromal compartments and often fails to identify the poor-prognostic CMS4 mesenchymal group in immortalized cell lines and patient-derived organoids. CMScaller uses cancer cell-intrinsic, subtype-specific gene expression markers as features for *Nearest Template Prediction* [2].

2 Input data

`CMScaller` provides robust *cross platform and sample-type* performance given a balanced, homogeneous dataset of >40 unique samples. For less than ~40 samples, sampling variance (by-chance subtype depletion/enrichment) is a concern. Similarly, selection, *e.g.* excluding microsatellite instable (MSI) samples or including only aggressive cancers, would break an underlying assumption and bias the resulting predictions [3].

3 Quick start

3.1 Installation and dependencies

The following packages are required in order to run examples in this vignette.

- Bioconductor[4]: *Biobase*, *limma*

In addition, *edgeR* is needed for specific RNA-sequencing normalization methods and *parallel*, *snow* for `ntp` parallelization.

```
# dependencies: run if not already installed
source("https://bioconductor.org/biocLite.R")
biocLite(c("Biobase", "limma"))
# proper repository to be fixed for publication
install.packages("pathToPackageFile/CMScaller_0.99.0.tar.gz", repos=NULL)
```

3.2 CMS classification

`CMScaller` function requires an expression matrix or ExpressionSet as input (`emat`). Gene names in `rownames(emat)` must be NCBI Entrez, Ensembl or HGNC symbol identifiers. For gene symbols or Ensembl identifiers, parameter `rowNames` must be set to `symbol` or `ensg`, respectively. The code chunk below demonstrates how to perform classification using TCGA primary colorectal cancer example data [5].

- microarray data input should be pre-processed and normalized (often log_2transformed)[1].
- RNA-sequencing counts/RSEM values could be used directly by setting `RNAseq=TRUE` which activates quantile normalization and log_2transform.

```
library(Biobase) # if input is ExpressionSet
library(CMScaller)
# get RNA-seq counts from TCGA example data
counts <- exprs(crcTCGAsubset)
head(counts[,1:3])
##           TCGA-4N-A93T-01A-11R TCGA-4T-AA8H-01A-11R TCGA-5M-AAT4-01A-11R
## 100133144                    9                   13                   18
## 10431                     2450                 1215                 2976
## 57714                      375                  536                  961
## 645851                      43                   18                   29
## 652919                       0                    0                    0
## 729884                       0                    2                    3
# prediction and gene set analysis
par(mfrow=c(1,2))
res <- CMScaller(emat=counts, RNAseq=TRUE, FDR=0.1)
## performing log2-transform and quantile normalization...
## cosine correlation distance
## 92 samples; 4 classes; 82-237 features/class
## serial processing; 1000 permutation(s)...
## predicted samples/class (FDR<0.05)
##
## CMS1 CMS2 CMS3 CMS4 <NA>
##   14   23   15   28   12
## 11/92 samples set to NA
cam <- CMSgsa(emat=crcTCGAsubset, class=res$prediction,RNAseq=TRUE)
## 11 samples with class or batch NA's excluded
```

```
# comparison with true class
table(pred=res$prediction, true=crcTCGAsubset$CMS)
##       true
## pred   CMS1 CMS2 CMS3 CMS4
##   CMS1    8    0    3    0
##   CMS2    0   20    0    0
##   CMS3    0    1    6    0
##   CMS4    0    2    0   19
head(res, n=3)
##                      prediction d.CMS1 d.CMS2 d.CMS3 d.CMS4 p.value    FDR
## TCGA-4N-A93T-01A-11R       <NA>   0.76   0.71   0.72   0.89   0.935 0.9558
## TCGA-4T-AA8H-01A-11R       CMS3   0.76   0.69   0.63   0.90   0.001 0.0014
## TCGA-5M-AAT4-01A-11R       <NA>   0.74   0.69   0.73   0.83   0.799 0.8355
```

- `rownames(res)` equals `colnames(emat)`
- class predictions with `NA` for samples with adjusted-p-value $>$ threshold

[1] Hoshida[2] does not explicitly state whether input should be log_2transformed or not and example data includes both. Such transformation reduces the weight of genes with large deviations and will usually affect results at the margins.

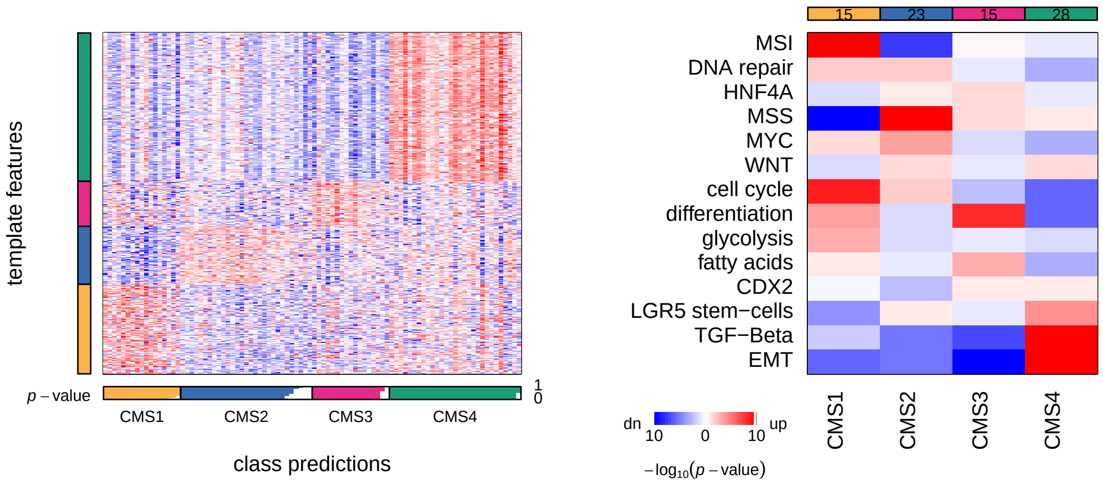

Figure 1: CMScaller graphic output. **Left** heatmap shows relative expression for template genes. Samples (columns) are ordered according to class predictions and confidence. The height of the white bars below gives the unadjusted prediction *p*-values. Genes (rows) are ordered according to class template. Heatmap color saturation indicates magnitude while red and blue indicate genes up and down relative to the sample mean. **Right** heatmap shows results for Camera gene set analysis. Heatmap color saturation indicates statistical significance and red and blue indicates direction of change.

- templates distances
- prediction p-values[2]
- prediction FDR-adjusted p-values

4 Package details

`CMScaller` is basically a wrapper function for `ntp`. Similarly, `CMSgsa` just provides some presets for `subCamera`.

4.1 Preparing custom templates

Templates consists of sets of subtype-specific marker genes. `subDEG` performs *limma* differential expression analysis for identification of such markers. Below, is an example on how to prepare custom templates based on a training set with known class labels. `doVoom=TRUE` enables voom transformation - required for proper *limma* modeling of RNA-sequencing counts [6].

```
emat <- crcTCGAsubset
cms <- emat$CMS.Syn
train <- sample(seq_along(cms), size=length(cms)/(2))
deg <- subDEG(emat[,train], class=cms[train], doVoom=TRUE)
## 11 samples with class or batch NA's excluded
templates <- ntpMakeTemplates(deg, resDEG=TRUE, topN=50)
templates$symbol <- fromTo(templates$probe)
tail(templates,n=3)
##     probe class symbol
## 198 85360  CMS4  SYDE1
```

[2]lowest possible estimate of the p-value is 1/permutations

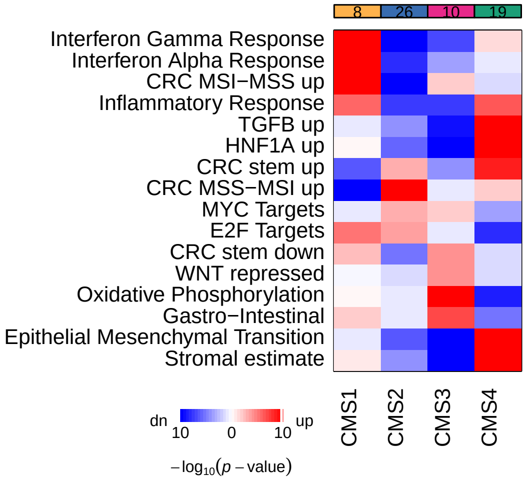

Figure 2: Gene Set Analysis (GSA) shows that CMS are biologically distinct.

```
## 199  2213  CMS4 FCGR2B
## 200  7058  CMS4  THBS2
```

4.2 Gene Set Analysis

subCamera provides gene set analysis and visualization and is a wrapper functions for camera in the *limma* package. camera controls for intra-set gene-wise correlations in order to reduce false-positive rate while retaining statistical power [7, 8]. CMSgsa provides preset gene sets to subCamera.

```
# increase left margins to accomodate gene set names
par.old <- par()
par(mfrow=c(1,1), mar=par.old$mar+c(0,4,0,0))
subCamera(emat, cms, geneList=geneSets.CRC, doVoom=TRUE)
## 29 samples with class or batch NA's excluded
```

```
# restore margins
par(mar=par.old$mar)
```

4.3 Nearest Template Prediction

ntp matches templates$probe against rownames(emat). Missing features and features with NA/NaN's are ignored in the prediction. emat should be row-wise centered and scaled.

```
# loads included emat, scales and centers
emat <- crcTCGAsubset
emat_sc <- ematAdjust(emat, normMethod="quantile")
head(emat_sc[,1:3])
##           TCGA-4N-A93T-01A-11R TCGA-4T-AA8H-01A-11R TCGA-5M-AAT4-01A-11R
## 100133144                -0.30                 0.59                 0.16
## 10431                     0.80                -0.17                 1.05
## 57714                    -0.90                 0.17                 0.11
```

```
## 645851                          1.71                1.39                1.05
## 652919                         -0.99               -0.85               -1.04
## 729884                         -0.77                0.59                0.40
```

ntp function requires an expression matrix and templates. Since prediction confidence is estimated from permutations, strict p-value reproducibility requires set.seed.

```
# test set prediction
res <- ntp(emat_sc[,-train], templates, nPerm=1000)
res <- subSetNA(res, pValue=.1)
## 3/46 samples set to NA
table(pred=res$prediction, true=cms[-train])
##       true
## pred   CMS1 CMS2 CMS3 CMS4
##   CMS1    2    0    2    0
##   CMS2    0    8    0    1
##   CMS3    1    3    1    0
##   CMS4    2    0    0    8
head(res)
##                         prediction d.CMS1 d.CMS2 d.CMS3 d.CMS4 p.value    FDR
## TCGA-4N-A93T-01A-11R          CMS3   0.68   0.71   0.63   0.92   0.012 0.0131
## TCGA-5M-AAT4-01A-11R          <NA>   0.68   0.72   0.73   0.85   0.639 0.6536
## TCGA-A6-5657-01A-01R          CMS4   0.78   0.79   0.79   0.53   0.001 0.0012
## TCGA-A6-5664-01A-21R          CMS4   0.72   0.75   0.66   0.53   0.001 0.0012
## TCGA-A6-5667-01A-21R          CMS2   0.80   0.56   0.83   0.68   0.001 0.0012
## TCGA-A6-6137-01A-11R          CMS3   0.70   0.63   0.59   0.84   0.001 0.0012
```

ntp output is a data.frame with $3 + K$ columns where K is the number of classes. Rows represent columns in input emat.

- rownames(res) equals colnames(emat)
- class predictions with levels(res$prediction) equaling levels(templats$class)
- templates distances (defaults to cosine correlation distance)
- prediction p-values
- prediction FDR-adjusted p-values

subSetNA function resets predictions with p-value or FDR above some arbitrary threshold to NA.

5 Nearest Template Prediction

Nearest template prediction (NTP) was proposed as a classification algorithm by Yujin Hoshida and published in *PLoS ONE* in 2010 [2]. It aims to provide robust single-sample class prediction for high-dimensional, noisy gene expression data. In brief, first, for each subclass, *a template*, a list of genes coherently upregulated is determined. Then, for each sample, *the distance* to each template is calculated and class is assigned based on the smallest distance. Finally, prediction confidence is assessed based on the distance of the null-distribution, estimated from *permutation tests* (feature permutation). The default distance metric selected by Hoshida was a cosine similarity-derived distance (see below). When applied to a reasonably well-balanced homogeneous dataset, row-wise centering and scaling is performed (gene means and standard deviations $\mu = 0$, $\sigma = 1$). In case of single-sample prediction, feature-wise means and standard deviations from a previous sample set are used to perform sample-wise scaling and centering. The key advantages of the NTP algorithm are conceptual simplicity, biological plausibility, ease of implementation and robustness.

Formally, N samples with expression values for P genes divided into K different classes.

- $\mathbf{X}_{[P,N]}$ centered and scaled expression matrix where column vector $x_{[P]}$ is the expression for sample n.

- M is a list of K vectors where each element m is a set of marker features with higher expression in samples belonging to class k as compared to remaining samples. m's may be of uneven length, but are typically $<<P$
- $\mathbf{Y}_{[P,K]}$ template matrix where $y_{[P]} = [p \in m]$ for class k (0 if not marker, 1 otherwise).

For the sample and template vectors x and y, a proper distance metric, $d_{x,y}$ for the similarity function $f(x,y)$ is given by $d = \sqrt{\frac{1}{2}(1 - f(x,y))}$ [9]. Here f is either cosine, Kendall, Pearson or Spearman correlation. Cosine similarity, the angle between two Euclidean vectors is given by

$$f(x,y) = \cos(\theta) = \frac{\sum xy}{\sqrt{\sum x^2}\sqrt{\sum y^2}}$$

The following code chunks demonstrate NTP in code.

```
# random centered/scaled expression matrix and templates
set.seed(42)
N <- 5000;P <- 5000;K <- 4;nPerm <- 1000;n <- 1
X <- matrix(rnorm(P*N, mean=0, sd=1), ncol=N)
Y <- matrix(rbinom(P*K, size = 1, prob=.01), ncol=K)
# sample-template correlations (implemented in corCosine)
cos.sim <- crossprod(X,Y) / outer(
                sqrt(apply(X, 2, crossprod)),
                sqrt(apply(Y, 2, crossprod)))
# sample-template distances (vectorized)
simToDist <- function(cos.sim) sqrt(1/2 * (1-cos.sim))
cos.dist <- simToDist(cos.sim)
hist(cos.dist, xlab="cosine correlation distance")
```

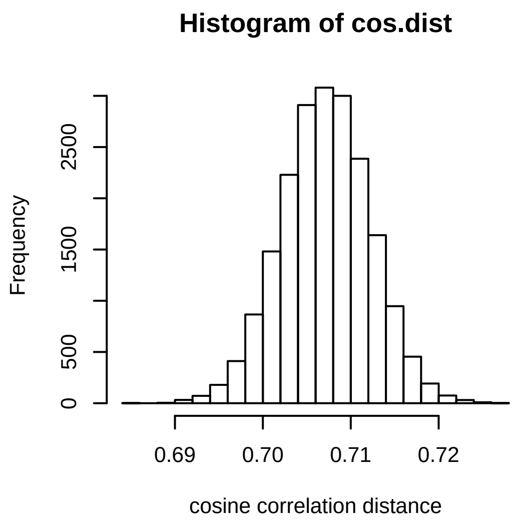

For centered, scaled and uncorrelated data, the cosine correlation distance is $\sqrt{0.5 \times (1-0)} \approx 0.707$.

Resulting distances is ranked among distances of permutated samples and used to estimate prediction confidence. The lowest possible p-value estimate is therefore $1/permutations$

```
# estimate prediction confidence
pred.class <- apply(cos.dist, 1, which.min)
pred.dists <- apply(cos.dist, 1, min)
null.dist <- replicate(nPerm, min(simToDist(corCosine(sample(X[,n]), Y))))
p <- rank(c(pred.dists[n], null.dist))[1]/(length(null.dist))
```

Code below is not evaluated for this vignette, but illustrates the uniform p-value distribution for centered and scaled uncorrelated input[3].

```
# rearrange matrix and templates for ntp input
rownames(X) <- make.names(seq_len(P))
templates <- lapply(seq_len(K), function(k) rownames(X)[Y[,k]==1])
names(templates) <- paste("k", seq_len(K))
templates <- ntpMakeTemplates(templates, resDEG = FALSE)
# takes a couple of minutes: 5000 samples and 1000 permutations
res <- ntp(X, templates, nCores=4L, nPerm=nPerm, doPlot=TRUE)
# expect uniform distribution
hist(res$p.value)
```

6 Session

```
## R version 3.4.2 (2017-09-28)
## Platform: x86_64-pc-linux-gnu (64-bit)
## Running under: Ubuntu 16.04.3 LTS
##
## Matrix products: default
## BLAS: /usr/lib/libblas/libblas.so.3.6.0
## LAPACK: /usr/lib/lapack/liblapack.so.3.6.0
##
## locale:
##  [1] LC_CTYPE=en_US.UTF-8       LC_NUMERIC=C
##  [3] LC_TIME=nb_NO.UTF-8        LC_COLLATE=C
##  [5] LC_MONETARY=nb_NO.UTF-8    LC_MESSAGES=en_US.UTF-8
##  [7] LC_PAPER=nb_NO.UTF-8       LC_NAME=C
##  [9] LC_ADDRESS=C               LC_TELEPHONE=C
## [11] LC_MEASUREMENT=nb_NO.UTF-8 LC_IDENTIFICATION=C
##
## attached base packages:
## [1] parallel  stats     graphics  grDevices utils     datasets  methods
## [8] base
##
## other attached packages:
## [1] CMScaller_0.99.1    Biobase_2.36.2      BiocGenerics_0.22.1
## [4] BiocStyle_2.4.1
##
## loaded via a namespace (and not attached):
##  [1] Rcpp_0.12.13    digest_0.6.12   rprojroot_1.2   backports_1.1.1
##  [5] magrittr_1.5    evaluate_0.10.1 highr_0.6       stringi_1.1.5
##  [9] limma_3.32.10   rmarkdown_1.6   tools_3.4.2     stringr_1.2.0
## [13] yaml_2.1.14     compiler_3.4.2  htmltools_0.3.6 knitr_1.17
```

References

1. Guinney J, Dienstmann R, Wang X, Reyniès A de, Schlicker A, Soneson C, Marisa L, Roepman P, Nyamundanda G, Angelino P, Bot BM, Morris JS, Simon IM, Gerster S, Fessler E, Melo FDSE, Missiaglia E, Ramay H, Barras D, Homicsko

[3] present NTP implementation provides more conservative p-value estimates than Hoshida[2].

K, Maru D, Manyam GC, Broom B, Boige V, Perez-Villamil B, Laderas T, Salazar R, Gray JW, Hanahan D, Tabernero J, et al.: **The consensus molecular subtypes of colorectal cancer**. *Nat Med* 2015, **21**:1350–1356.

2. Hoshida Y: **Nearest Template Prediction: A Single-Sample-Based Flexible Class Prediction with Confidence Assessment**. *PLoS One* 2010, **5**:e15543.

3. Zhao X, Rødland EA, Tibshirani R, Plevritis S: **Molecular subtyping for clinically defined breast cancer subgroups**. *Breast Cancer Res* 2015, **17**.

4. Huber W, Carey VJ, Gentleman R, Anders S, Carlson M, Carvalho BS, Bravo HC, Davis S, Gatto L, Girke T, Gottardo R, Hahne F, Hansen KD, Irizarry RA, Lawrence M, Love MI, MacDonald J, Obenchain V, Oleś AK, Pagès H, Reyes A, Shannon P, Smyth GK, Tenenbaum D, Waldron L, Morgan M: **Orchestrating high-throughput genomic analysis with Bioconductor**. *Nat Meth* 2015, **12**:115–121.

5. TCGA: **Comprehensive molecular characterization of human colon and rectal cancer**. *Nature* 2012, **487**:330–337.

6. Law CW, Chen Y, Shi W, Smyth GK: **Voom: Precision weights unlock linear model analysis tools for RNA-seq read counts**. *Genome Biology* 2014, **15**:R29.

7. Wu D, Smyth GK: **Camera: A competitive gene set test accounting for inter-gene correlation**. *Nucl Acids Res* 2012, **40**:e133.

8. Ritchie ME, Phipson B, Wu D, Hu Y, Law CW, Shi W, Smyth GK: **Limma powers differential expression analyses for RNA-sequencing and microarray studies**. *Nucl Acids Res* 2015, **43**:e47.

9. Dongen S van, Enright AJ: **Metric distances derived from cosine similarity and Pearson and Spearman correlations**. *arXiv:12083145 [cs, stat]* 2012.
